# Supplementary material for: Content-rich biological network constructed by mining PubMed abstracts
Source: BMC Bioinformatics. 2004 Oct 8;5:147. doi: 10.1186/1471-2105-5-147 (PMC528731; doi:10.1186/1471-2105-5-147)
Supplement: Additional File 5 — The original Chilibot query results of the term "long-term potentiation (LTP)" and 22 other terms, limiting the latest references analyzed to the years 1990, 1995, 2000, and 2004. [file 1471-2105-5-147-S5.bz2 › chilibotAdditionalFile5/ltp2004/html/TRKB_SYNAPTOTAGMIN.html]

 


 **TRKB** and **SYNAPTOTAGMIN** 
  
Found 2 abstracts in PubMed,  **2 abstracts were retrieved and analyzed**.  


---

 Search Google  |
 PDF files only 
|  EDU domain only 

---

- Neurochem Res, 2002   Cholinergic neurons of the nucleus basalis are selectively vulnerable in Alzheimer s disease , yet the molecular mechanisms associated with their dysfunction remain unknown.
  We used single cell RNA amplification and custom array technology to examine the expression of functional classes of mRNAs found in anterior NB neurons from normal aged and AD subjects.
  mRNAs encoding neurotrophin receptors, synaptic proteins, protein phosphatases, and amyloid related proteins were evaluated.
  We found that **trkB** and trkC mRNAs were selectively down regulated in NB neurons.
  . . . p75NTR mRNA levels remained stable in end stage AD.
  TrkA mRNA was reduced by approximately 28%, but did not reach statistical significance.
  There was a down regulation of synaptophysin, **synaptotagmin**, and protein phosphatases PP1alpha and PP1beta mRNAs in AD.
  In contrast, we found a selective up regulation of cathepsin D mRNA in NB neurons in AD brain.
  Thus, anterior NB neurons undergo selective alterations in gene expression in AD.
  These results may provide clues to the molecular pathogenesis of NB neuronal degeneration during AD.
